# FAM-MDR: A Flexible Family-Based Multifactor Dimensionality Reduction Technique to Detect Epistasis Using Related Individuals

**DOI:** 10.1371/journal.pone.0010304

**Published:** 2010-04-22

**Authors:** Tom Cattaert, Víctor Urrea, Adam C. Naj, Lizzy De Lobel, Vanessa De Wit, Mao Fu, Jestinah M. Mahachie John, Haiqing Shen, M. Luz Calle, Marylyn D. Ritchie, Todd L. Edwards, Kristel Van Steen

**Affiliations:** 1 Montefiore Institute, University of Liège, Liège, Belgium; 2 Groupe Interdisciplinaire de Génoprotéomique Appliquée - Research, University of Liège, Liège, Belgium; 3 Department of Systems Biology, University of Vic, Vic, Spain; 4 Miami Institute for Human Genomics, University of Miami, Miami, Florida, United States of America; 5 Department of Applied Mathematics and Computer Science, Ghent University, Ghent, Belgium; 6 School of Medicine, University of Maryland, Baltimore, Maryland, United States of America; 7 Center for Human Genetics Research, Vanderbilt University, Nashville, Tennessee, United States of America; VU University Medical Center and Center for Neurogenomics and Cognitive Research, VU University, Netherlands

## Abstract

We propose a novel multifactor dimensionality reduction method for epistasis detection in small or extended pedigrees, FAM-MDR. It combines features of the Genome-wide Rapid Association using Mixed Model And Regression approach (GRAMMAR) with Model-Based MDR (MB-MDR). We focus on continuous traits, although the method is general and can be used for outcomes of any type, including binary and censored traits. When comparing FAM-MDR with Pedigree-based Generalized MDR (PGMDR), which is a generalization of Multifactor Dimensionality Reduction (MDR) to continuous traits and related individuals, FAM-MDR was found to outperform PGMDR in terms of power, in most of the considered simulated scenarios. Additional simulations revealed that PGMDR does not appropriately deal with multiple testing and consequently gives rise to overly optimistic results. FAM-MDR adequately deals with multiple testing in epistasis screens and is in contrast rather conservative, by construction. Furthermore, simulations show that correcting for lower order (main) effects is of utmost importance when claiming epistasis. As Type 2 Diabetes Mellitus (T2DM) is a complex phenotype likely influenced by gene-gene interactions, we applied FAM-MDR to examine data on glucose area-under-the-curve (GAUC), an endophenotype of T2DM for which multiple independent genetic associations have been observed, in the Amish Family Diabetes Study (AFDS). This application reveals that FAM-MDR makes more efficient use of the available data than PGMDR and can deal with multi-generational pedigrees more easily. In conclusion, we have validated FAM-MDR and compared it to PGMDR, the current state-of-the-art MDR method for family data, using both simulations and a practical dataset. FAM-MDR is found to outperform PGMDR in that it handles the multiple testing issue more correctly, has increased power, and efficiently uses all available information.

## Introduction

The International HapMap Project [1] was designed to create a genome-wide database of human genetic variation, with the expectation that these data would be useful for genetic association studies of common diseases. This expectation has been fulfilled with just the initial output of genome-wide association analyses, identifying nearly 500 loci for over 80 common diseases and traits [2], [3]. Despite these successes, it has become clear that usually only a small percentage of total genetic heritability estimates can be explained by the identified loci. For instance, for inflammatory bowel disease (IBD), 32 loci significantly impact disease but they explain only 10% of disease risk and 20% of genetic risk [4]. This may be attributed to the fact that recent findings show many types of genetic associations for various traits, with subtle effects: non-additive genetic effects, non-SNP polymorphisms, epigenetic effects, but also gene-environment and gene-gene interactions [5].

The role of genetic interactions in explaining phenotypic variability has been described in several publications [6], [7], [8], [9], [10], [11], [12], [13], [14], [15]. Interactions may lead to inconsistent results from the masking of associations, they can be suggestive of important pathogenic mechanisms and may elucidate relevant opportunities for intervention [16], [17]. Epistasis, defined as the deviation from additivity of effects observed at multiple genetic exposures [18], [19], may also explain part of the genetic heritability that is left unexplained for most complex disorders [20]. These reasons have made epistasis an increasingly accepted characteristic of the genetic architecture of common, complex disorders [21], [22], [23], [24].

One of the potential reasons for the small number of large-scale genetic interaction studies performed in humans so far is that although genetic interactions identified from model organisms provide insight into biological processes, these biological processes often lack sufficient overlap with other types of gene/protein associations with traits of interest [25], [26]. Also, the relatively low success rate of large-scale epistasis searches to date may simply reflect the limited ability to assess the many possible modes of interaction, including pairwise interactions and threshold effects [27] or inadequate solutions given to a difficult statistical challenge [21]. In addition, subtle variation in allele frequency can either introduce an interaction effect and likewise remove an interaction effect from a particular dataset; this can make detection of epistasis effects quite challenging [28]. Overviews of methods for epistasis detection were given by Cordell [29], [30] and by Onkamo and Toivonen [31].

One non-parametric approach developed for epistasis analysis is Multifactor Dimensionality Reduction [32], [33], [34] (MDR). Since its conception, many methodological and applied papers have emerged that build on or use MDR. To our knowledge, the current state-of-the-art MDR-related method that can accommodate nuclear families of any size and different types of outcome variables is the recently proposed Pedigree-based Generalized MDR method [35] (PGMDR) which generalizes the Generalized MDR method [36] (GMDR) to family data. Its competitor, the MDR Pedigree Disequilibrium Test [37] (MDR-PDT), is only suited for case-control data and does not allow for covariate adjustments. Similar to MDR, GMDR uses prediction accuracy measures for best model selection. Significance assessment is based on random permutations. To easily accommodate continuous traits and variable adjustments, GMDR is based on scores of a (generalized) linear model. In the special case of no covariates and a binary outcome it reduces to the classical MDR. PGMDR first constructs a non-transmitted genotype for every non-founder in the pedigree. When parental genotype information is missing, PGMDR samples one realization of the nontransmitted genotype from the conditional distribution given the minimal sufficient statistic for the null hypothesis through an algorithm that is modified from Rabinowitz and Laird [38]. Second, the non-founders and the non-transmitted genotypes are analyzed by the GMDR algorithm. Significance assessment is again based on permutations. To maintain the correlation structure within the families, families as a whole are used as permuting units and the transmitted and non-transmitted sets in a whole family are randomly shuffled. PGMDR software is available from the URL http://www.healthsystem.virginia.edu/internet/addiction-genomics.

The need for new statistical methods to overcome some of the remaining statistical hurdles in epistasis detection, has led to the development of FAM-MDR. The method combines features of the GRAMMAR approach [39] with features of Model-based MDR [40] (MB-MDR).

In the Materials and Methods section, we introduce the FAM-MDR algorithm and describe our extensive simulation study to examine type I error and power of this approach. To examine the application of FAM-MDR to determine epistasis in family studies of a complex disease, we examined data on glucose area-under-the-curve (GAUC), an endophenotype of Type 2 Diabetes Mellitus (T2DM) for which multiple independent genetic associations have been observed, in the extended pedigrees of the Amish Family Diabetes Study [41] (AFDS). Subsequently, we describe the power and type I error performance of FAM-MDR in our simulations and application to AFDS, as well as a comparative study between FAM-MDR and PGDMR, in the Results section. Finally, the Discussion elaborates on the significance of our results, and the relevance of its application to finding gene-gene interactions in a complex disease like T2DM.

## Materials and Methods

### The FAM-MDR algorithm

FAM-MDR is an acronym for FAMily Multifactor Dimensionality Reduction and is an adaptation to related individuals of the Model-Based Multifactor Dimensionality Reduction method [40] (MB-MDR) for epistasis detection with unrelated individuals. An implementation of the FAM-MDR algorithm is available through the URL www.statgen.be. The approach consists of two parts.

Part I: In order to deal with familial correlations between observations, data are first analyzed using a polygenic model
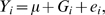
(1)with 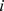 indexing individuals, 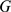 distributed 
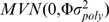
 and 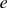 distributed 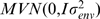, representing the additive polygenic and environmental effects respectively. The polygenic effect has variance 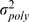 and is correlated within families, with correlation matrix equal to the relationship matrix 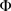. For the calculations we use the polygenic function of R package GenABEL [39], [42], [43], [44]. This package can be retrieved from the URL http://cran.r-project.org/web/packages/GenABEL/index.html. The relationship matrix 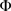 can be derived either theoretically from the pedigree structure, or can be estimated from the available genomic data, in which case the genomic kinship is computed [45]. Genomic kinship is to be preferred when genome-wide data are available, but of course in a candidate gene study genomic kinship cannot be estimated in a reliable way and one will have to use pedigree kinship [Aulchenko, GenABEL Tutorial]. The environmental variance 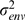 is assumed to be the same for all individuals. In addition, environmental effects are assumed to be independent between individuals, whether belonging to different families or within the same family, giving rise to an environmental variance-covariance matrix 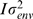 (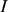: the identiy matrix of rank equal to the number of individuals).

The residuals
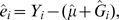
(2)that have been derived from (1), are free from polygenic familial correlation and can serve as new familial correlation-free traits to be used in a genetic association analysis with measured genetic markers. As a remark, 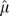 and 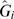 are estimates of mean 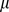 and polygenic effect (or breeding value) 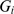 obtained by the Expectation-Maximisation (EM) algorithm using the maximum likelihood (ML) paradigm, hence maximizing the joint likelihood of fixed effects and variance components [44]. In the GRAMMAR approach of Aulchenko et al. [39], such a genetic association analysis targets associations with single markers, one at a time, and is fully parametric in nature. In contrast, in the FAM-MDR approach, associations with multiple loci at once are evaluated.

**Figure 1:**
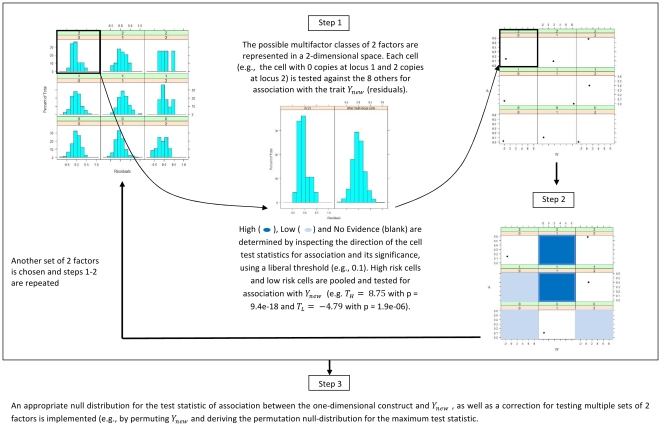
Summary of the steps involved in a FAM-MDR analysis. The figure shows the three steps of FAM-MDR Part II on one of the simulated datasets for Model M27, 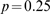, 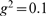 and 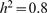, for the analysis without main effects correction.

Part II: Once the data have been prepared in Part I, FAM-MDR proceeds with investigating the association between the newly defined trait 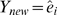 from Part I (in particular the aforementioned residuals) and multi-locus measured genotypes, using the potentially fully-parametric MB-MDR method (Figure 1). It is justified to apply MB-MDR, that was developed for unrelated individuals, to these residuals. Indeed, conditional on the observed genotypes all the familial correlation has been accounted for. Moreover, MB-MDR flexibly deals with different outcome types, including those measured on a continuous scale. Like MDR, MB-MDR reduces a high-dimensional interaction space to a 1-dimensional space, by assigning genetic effect size labels to multi-locus genotypes, which will be further referred to as risk labels.

Key steps involved in a FAM-MDR Part II analysis are summarized graphically in Figure 1. Because some characteristics of MB-MDR have been altered in the FAM-MDR algorithm, we summarize the key steps and properties of MB-MDR, and point out changes we made to the initial MB-MDR algorithm. Throughout this paper, for ease of exposition, we focus on two-locus models and diallelic markers, although both MB-MDR and FAM-MDR are applicable in principle to more general settings as well.

In MB-MDR step 1, for a selection of a pair of SNPs, each genotype cell is tested against the eight others for association with the trait. The cells that are found to be significantly associated with the outcome, at a liberal threshold of 0.1, are then called high risk (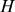) or low risk (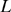) based on the position of the selected association measure (e.g. 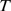 statistic or odds ratio) in the spectrum of all possibilities. Those cells that are not significant at the threshold 0.1 are labeled as non-evidence cells (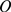). For a more detailed discussion of the MB-MDR method we refer to Calle et al. [40] and also to their technical report available from the URL http://www.recercat.net/handle/2072/5001.

In step 2, two additional tests are performed for association with the trait: testing 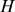 versus 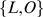 and testing 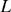 versus 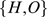. This gives rise to two Wald-type statistics of association, 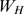 and 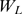, that are either derived from a parametric or a non-parametric testing approach.

In step 3, the significance of 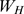 and 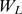 is assessed through permutations. This is different from the classical MB-MDR implementation, but is an elegant way to compensate for the data snooping in MB-MDR steps 1–2 and to correct for the otherwise overly optimistic test results. Note that in the initial implementation of MB-MDR, depending on the number of combined cells in either the high (low) risk cells pool, a different null distribution for the corresponding Wald test statistic 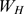 (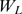) was derived. These marginal null distributions were generated by simulating reference data with similar characteristics. The multiple testing issue that arises when considering different SNP pair combinations, is tackled by MB-MDR [40] using BH-FDR (Benjamini-Hochberg False Discovery Rate) methodology.[46] Because of the aforementioned dependency on number of combined cells, and the complexity of the significance assessment via a simulation-based null distribution derivation in step 3, we chose to implement a different approach. FAM-MDR therefore implements a permutation strategy by simply randomly permuting the familial correlation-free residuals (2) obtained in FAM-MDR Part I. This assumes a general null-hypothesis of no association between any of the measured markers and trait (no main effects, no interaction effects). This strategy does not depend on the number of combined cells, nor on reference data.Although MB-MDR allows in principle for multiple model selection, we have furthermore restricted the approach to select only the best model, for the purpose of comparison with PGMDR. In particular, we derive the permutation null distribution of the maximum test statistic, i.e. maximized over the two different tests (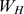 and 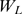) and all two-SNP combinations 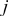: 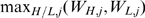 and recommend 1000 replications for doing so.

Moreover, we emphasize that corrections for main effects and covariate adjustments are also possible in FAM-MDR and we choose to carry these out prior to association testing. Extending the polygenic model (1) to a (two-locus) measured genotype (MG) model [47] with the particular main effect/covariates present in the model, has several advantages over adjusting while identifying risk cells and/or testing for association between new one-dimensional genetic constructs and the trait of interest. First, it leads to more stable parametric estimations when adopting a parametric regression approach in FAM-MDR Part II. Second, a priori adjustment paves the way for a fully non-parametric epistasis screening and thus faster MB-MDR runs on adjusted residuals. Nevertheless, for the purpose of this paper, we will continue to use the parametric linear model paradigm (in fact the one-parameter Wald test is equivalent to the Student t-test) and not the non-parametric alternative, the Wilcoxon rank sum test. Third and most importantly, when residuals are free from identified important main effects, the null hypotheses of no association and of no epistasis become equivalent and the permutation procedure is a correct procedure for claiming epistasis, i.e. association above and beyond the main effects corrected for in FAM-MDR Part I.

Finally, a note on how FAM-MDR handles missing data. For missing genotypes, FAM-MDR uses the available cases (AC) paradigm. In other words, if a particular pair is considered, only individuals with complete data for this pair are included in the analysis, while individuals with missing data for one or two SNPs involved are not considered. Individuals with missing trait values are used in FAM-MDR Part I only when they are useful for deriving the relationship matrix, but never for the polygenic calculations themselves. Indeed, if the number of markers is small, the theoretical relationship matrix is used and the complete pedigree is needed for calculating this in a correct way. When the number of markers is large, FAM-MDR relies on the genomic kinship matrix and there is no need to keep individuals with missing trait values in the analysis. Note that since we are not using the individuals with missing trait values for the polygenic calculations, we essentially are performing a complete case (CC) analysis. Also, when correcting for main effects/covariates - since we only use those individuals with complete data on the main effects/covariates that are regressed out in Part I, we are again following the CC paradigm. In Part II, FAM-MDR obviously does not use missing new trait values, whether these are due to missing trait values or missing main effects/covariates adjusted for. Missing new trait values are also not included in the permutation procedure. It is good to recall that both AC and CC are valid under a missing completely at random (MCAR) missingness process [48].

### Simulation study

We simulated data consisting of 250 nuclear families, with the number of children drawn from a multinomial distribution with probabilities 1/4 to have one child (hence a trio), 1/2 to have two children, and 1/4 to have three children. On average, this gave rise to 1000 individuals. We assumed that no data were missing. In other words, complete information on genotypes at all loci and complete information on phenotypes were available.

To generate genotypes for these individuals, we first generated ten diallelic markers: 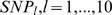, in linkage equilibrium. In addition, we assumed Hardy-Weinberg equilibrium for every generated marker. In other words, the genotype frequencies can be determined from the allele frequencies as follows: 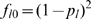, 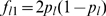, and 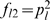. The allele frequencies of a non-functional SNP, 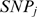 was fixed at 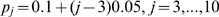, whereas the allele frequencies of the functional SNP pair 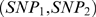 were taken to be equal, and varied as 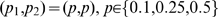. Parental genotypes were then drawn according to the population genotype frequencies above. Children's genotypes were assumed to follow Mendelian inheritance patterns.

Assuming the presence of additive polygenes and a residual environmental effect, phenotypes were simulated according to the (two-locus) MG model
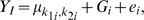
(3)with the notations of Expression (1) before. Note the difference of the fixed effect mean term, 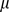 in (1), which is replaced by 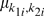 in (3). Here, 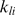 refers to the minor allele count 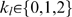 for individual 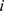 and 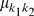 represents the mean trait values according to the functional SNP pair. Trait values per family were then sampled from a multivariate normal distribution, with as components of the mean vector the trait mean values 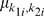 corresponding to the two-locus genotypes that the individuals constituting the family belong to, and as variance-covariance matrix the part of 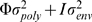 pertaining to that family. In what follows, we explain in detail the simulation parameter settings of our choice.

First, we notice that the population two-locus model variance 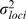 is computed as a weighted sum of squares, with weights determined by population genotype frequencies (exploiting no LD between the markers)
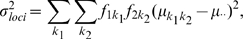
(4)in which the overall mean 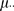 is computed as
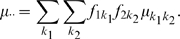
(5)In a similar way, the single-locus means and variances are respectively given by
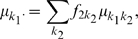
(6)

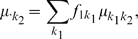
(7)and
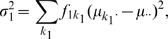
(8)

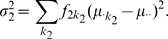
(9)Hence, since 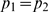, and therefore also 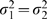, the epistatic variance 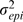, defined as the part of the two-locus model variance that is not explained by the contributing loci separately, is given in our simulation settings by 
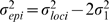
. Different contributions to the two-locus model variance of the epistatic variance 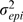 or the main effects model variance will be important in interpreting simulation results.

The total phenotypic variance, which is given by
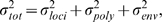
(10)was kept fixed at 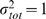 for all simulations. The total heritability 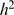 of the trait, defined as
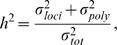
(11)was taken to be 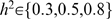. The proportion 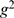 of the total variance 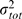 explained by the total two-locus model variance 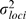,
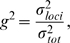
(12)varied as 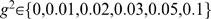. Because of the aforementioned assumptions, and the definitions for 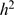 and 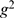, we have 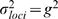, 
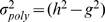
, and 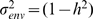. Hence, given 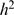 and 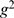, the three variance contributions are completely determined. Since only 10 SNPs were considered in all scenarios, the relationship matrix was derived from the nuclear family actual relationships.

Second, the fixed mean part in (3) needs to be determined. For this, we consider two two-locus models, M27 and M170, of Li and Reich [49], [50]. The key feature of Model M27 is that phenotypic means increase when for both loci at least one variant allele is present. In contrast, in Model M170 increased phenotypic values occur only when at one (and only one) locus a heterozygous genotype is observed. Explicitly, Models M27 and M170 are respectively determined by
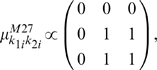
(13)And
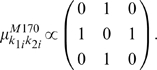
(14)It is important to realize that the two-locus variance 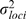, can be computed from the genotype-specific means 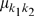 above and the minor allele frequency 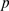. Hence, since the total trait variance 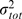 was set to 1, and since particular values for the two-locus model heritability 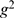 were also pre-specified, the means in (13) and (14) are actually proportional to the specifications to the right of the corresponding expression.

To enhance the interpretation of future simulation results, it is instructive to more closely look into the decomposition of the two-locus model variances for Models M27 and M170 into main effects and epistatic variance (Table 1). We observe that for Model M27 the contribution of main effects becomes increasingly important with increasing 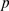, whereas for Model M170 the reverse is true, leading to a pure epistasis scenario for 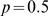.

**Table 1:** Relative importance of main effects and epistatic variances for different two-locus models.

	M27		M170	
*p*	Main	Epist.	Main	Epist.
0.10	0.16	0.68	0.29	0.42
0.25	0.30	0.39	0.06	0.88
0.50	0.43	0.14	0.00	1.00

Abbreviations: Main = ratio 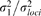 of each of the main effects variances 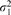 to the two-locus variance 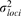, Epist. = ratio 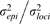 of the epistatic variance 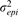 to the two-locus variance 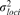.

The two different genetic two-locus models (M27 and M170), three possible minor allele frequencies 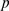 of the causal SNP pair, 5 different non-zero values for 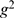 and three different values for the total heritability 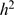, lead to a total of 90 simulation settings. For each of these settings 100 data sets were simulated.

Under the general null hypothesis of no association with the trait (no main effects, nor two-way interaction effects), in particular, 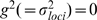, the different genetic models M27 and M170 are irrelevant. The three possible choices for the minor allele frequency 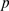 of the causal SNP pair, and the three possible values for 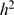 result in nine different general null hypothesis simulation settings. For each of these settings we generated 400 datasets.

Because we are primarily interested in detecting epistasis effects above and beyond main effects, when these main effects are present, we also generated a different type of null data, under the null hypothesis of no epistasis, but main effects. Hence, 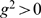 and
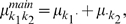
(15)effectively reducing the two-locus variance to 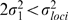, while increasing the polygenic variance. For each of the 75 simulation settings, we again generated 100 datasets.

To reduce the computational burden of our extensive simulation study, we adopted a sequential approach [51] during the permutation step of FAM-MDR (FAM-MDR Part II step 3). We carried out the permutation calculations in batches of 100, and each time evaluated a go or no go to continue or not, setting the maximum number of permutations to 1000. To this end, we determined the binomial 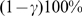 confidence interval of the significance level 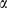 at the current number of permutations, with 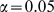 and 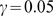. If the current estimate of the permutation p-value fell outside this confidence interval, the permutation procedure terminated and a definite conclusion about significance or non-significance was made. If the current estimate of the permutation p-value fell inside the confidence interval, no decisions were made about significance and an additional batch of 100 permutations were performed. A drawback of this sequential approach is that no accurate estimate of the permutation p-value is output. Hence, whereas it serves its purpose for simulation studies, it is less well suited for real data applications.

Only to enhance an honest comparison of simulations results with PGMDR, we also introduced a liberal version of FAM-MDR, referred to as FAM-MDR*. In contrast to PGMDR [35] and GMDR [36], for which the permutation null distribution is only derived with respect to the actual SNP pair identified by the initial epistasis screening, FAM-MDR screens all SNP pairs in a permuted dataset. By doing so, FAM-MDR assesses significance of the best SNP-pair, while appropriately correcting for multiple testing. FAM-MDR* assesses marginal significance of the best model by computing the empirical marginal p-value of the pair found. This was done by only maximizing over the two test statistics 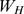 and 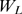, and by restricting attention to the specific pair 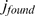 identified via the initial epistasis data analysis: 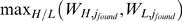. Although FAM-MDR* is less computationally intensive, it leads to overly optimistic results, and increased false positive rates, just like PGMDR. In line with PGMDR [35], 1000 permuations were used to estimate significance, hence no sequential approach is adopted.

Finally, we analyzed all simulated datasets with both FAM-MDR and FAM-MDR*. We applied PGMDR to all simulation settings with non-zero epistasis, and to 200 (out of 400) generated data sets for each of the simulations settings under the general null hypothesis of no association. Every analysis is carried out once with and without main effects correction. The former involves adjusting for SNP1 and SNP2 by assuming a co-dominant mode of inheritance. The analysis with correction for main effects leads to the detection of two-locus effects beyond co-dominant main effects. The analysis without correction for main effects leads to the detection of a two-locus model but without giving a clue about whether this model is genuinely indicative for epistasis or for main effects, or for both. In PGMDR analyses, and when correcting for SNP1 and SNP2 main effects, we created two dummy variables for each of the causal SNPs and submitted them as covariates to the PGMDR software.

### Amish Family Diabetes Study

The Amish Family Diabetes Study [41] (AFDS) is a study to identify the genetic determinants of type 2 diabetes and related traits in multi-generational extended pedigrees from the Old Order Amish (OOA) community, a genetically-isolated group in Lancaster County, Pennsylvania. For this analysis, we examined genotype data for 25 SNPs in five diabetes candidate genes, adipocnectin receptors 1 and 2 (*ADIPOR1*, *ADIPOR2*), adiponectin (*APM1*, also known as *ADIPOQ*), calsequestrin 1 (*CASQ1*), and hepatocyte nuclear factor 4A (*HNF4A*), in 1427 individuals from a single large multi-generational pedigree subdivided into 243 independent families for analysis and 25 SNPs. In previous analyses in the AFDS [52], [53], [54], [55], *rs1884614* in *HNF4A* [52], *rs2275703* and *rs617698* in *CASQ1* [54], and *rs1029629* in *ADIPOR2* [53] were found to be significantly associated with the continuous trait Glucose area under curve (GAUC). The latter was estimated using the trapezoid method from glucose levels taken at 30-minute intervals in a three-hour oral glucose tolerance test (OGTT) administered to all AFDS subjects without a prior history of diabetes [56]. We examined 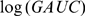 as our continuous trait of interest, in non-diabetic individuals with GAUC measurement available, setting 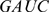 to missing for diabetics and individuals with unknown diabetes status because for diabetics the GAUC measurement is expected to be biased. We took the natural logarithm of 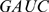 because the Shapiro-Wilk test strongly rejected normality of the residuals of the polygenic model in an analysis on 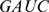 itself, whereas it did not so in an analysis on 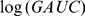. After removing Mendelian errors as found by FBAT (downloadable from URL http://www.biostat.harvard.edu/~fbat/default.html), we used the check.marker function of R package GenABEL on these 725 non-diabetics with GAUC measurement available. We put extr.call and extr.perid.call equal to 0.25, so that markers and individuals with call rates below 0.25 are removed prior to main analysis. For the main analysis we put callrate and perid.call to 0.9 so we discard iteratively markers and individuals with call rates below 0.9. We discarded four SNPs, *rs6666089* and *rs1342387* in *ADIPOR1*, and *rs617599* and *rs1186694* in *CASQ1*, due to low callrates. All markers had minor allele frequencies above 1%. We also discarded 105 individuals due to low callrates, by putting their GAUC measurement to missing. All analyses are based on the remaining 21 markers and 620 individuals.

In order to derive the correct relationships between the 620 individuals of interest, FAM-MDR also makes use of the remaining 807 individuals without GAUC measurement available and – in order to recognize relationships between offspring – of additional parental information (804 parents, without genotype nor phenotype information). Hence, “information” of 2231 individuals was exploited by FAM-MDR. The 620 individuals of interest correspond to 136 independent families. Of these, 75 consisted of unrelated single individuals with no genotyped first-degree relatives, 33 were nuclear families (comprising 51 of the 620 individuals or interest), and 28 were multi-generational families. However, these 28 families included 471 or 76% of the 620 individuals.

Because the present PGMDR implementation cannot deal with large multi-generational (non-nuclear) pedigrees, and only for reasons of comparison between PGMDR and FAM-MDR, we split multi-generational pedigrees into nuclear families. In the resulting set of nuclear pedigrees, 344 individuals were represented twice, most often as offspring in one pedigree and parents in another. The net result of the splitting process was a set of 584 non-independent nuclear families, comprising 2575 ( = 2231+344) individuals. Of the 620 individuals with GAUC measurement available, 161 were represented twice, resulting in a total of 781 individuals of interest to us, in 363 non-independent families. Of these families, 255 were part of the 28 extended pedigrees, while the remaining 108 were mutually independent. There is clearly a need to account for the lack of independence between these nuclear families. Unfortunately, the PGMDR software is unable to do so. Solely for reasons of comparison, FAM-MDR was applied to the split-pedigree data, as well as to the original multi-generational family data.

Analyses were carried out with and without correction for the main effects of *rs1884614* in *HNF4A*, *rs2275703* and *rs617698* in *CASQ1*, and *rs1029629* in *ADIPOR2*. They were regressed out in FAM-MDR Part I, or entered as covariates in PGMDR, using co-dominant coding. P-values were based on 1000 permutations for both PGMDR and FAM-MDR, hence no stopping rules were used. The significance level was set at 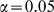.

Without main effects correction, FAM-MDR on the original data uses all 620 individuals with GAUC value available. When correcting for main effects, missing genotype information contributes to an increased missingness rate for the new trait values. Here only 572 or 92% of the 620 individuals have non-missing new trait values. On the split-pedigree data, 781 individuals are available for FAM-MDR without main effects correction, and 726 for FAM-MDR with main effects correction. Note that this is in fact an artificial increase in sample size of 26% and 27% respectively. In contrast to FAM-MDR, PGMDR works with the transmitted genotype information. Of the 620 individuals with a GAUC value available, the 75 unrelated individuals (singletons) obviously did not have parental information available, giving rise to 545 individuals eligible for PGMDR analysis. Furthermore, whenever genotypes for any SNP are completely missing for a family, PGMDR simply discards the whole family from the entire epistasis screening. As a result, PGMDR uses only 537 individuals in an analysis without correction for main effects, and 501 individuals in an analysis with main effects correction, amounting to reductions of 13% and 12% compared to the corresponding FAM-MDR analyses. Consequently, PGMDR does not use all information available.

## Results

### Simulation study

As a quality check on the simulated data, we first assessed the values of the Hardy Weinberg and Linkage Disequilibrium correlation coefficients 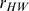 and 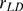, in the parents only. All correlation coefficients were consistent with the theoretical value of 0. More specifically, we observed that for all considered simulation settings the 95% confidence intervals of the correlation coefficients 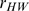 and 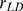 lied within the range 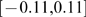. We have also fit a full MG model with a covariate coding for the exact dichotomization that is behind our models. In this way we were able to consistently estimate the effect sizes, showing that our data are indeed behaving in the way we intended.

**Figure 2:**
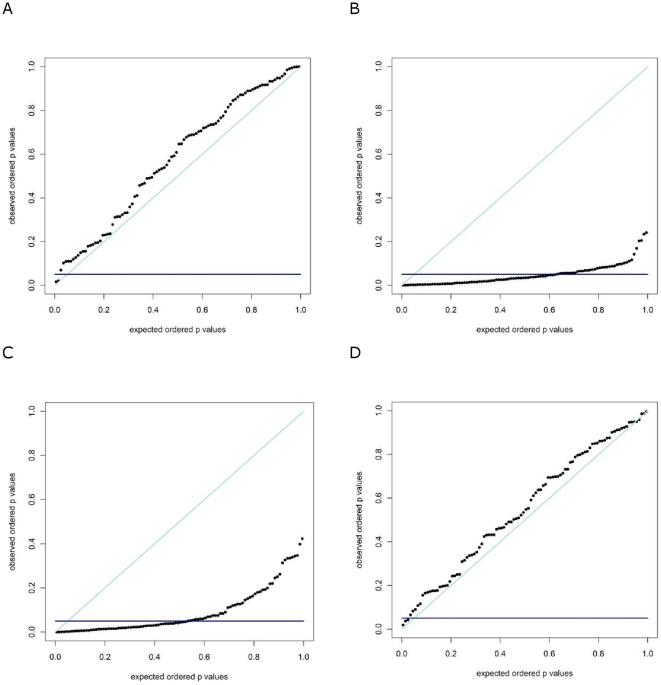
Probability-probability plots based on 100 replicates under the complete null hypothesis of no association. Data are generated with no main effects and no two-way interaction effects, with 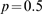 and 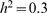. Analyses are performed without correction for main effects. The first three panels show results of FAM-MDR (A), FAM-MDR* (B) and PGMDR (C) for the usual situation study considering 10 SNPs. The final panel (D) shows PGMDR results when only 2 SNPs are considered. The straight lines indicate the theoretical probability-probability curve (light blue) and the 5% significance level (dark blue).

We first considered simulation results under the general null hypothesis of no association (no main effects, no two-order interactions). Empirical type I error rates were defined as the number of times the selected best 2-locus model was assessed significant, divided by the number of simulations. From Table 2, we notice that FAM-MDR typically gives smaller empirical type I error rates than the nominal type I error rate of 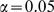. In other words, FAM-MDR has the tendency to be conservative. In contrast, FAM-MDR* and PGMDR are much too liberal. The latter is not surprising, since both FAM-MDR* and PGMDR completely ignore the multiple testing issue. These results are confirmed by Figure 2, showing the Probability-Probability (PP) plots of the p-value distributions under the general null hypothesis of no association for 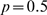 and 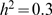. The PP-plots are based on 100 datasets and 1000 permutations, without stopping rule to obtain exact p-values. The plots show the expected ordered p-values 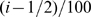 on the horizontal axis and the observed ordered p-values 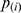 on the vertical axis. By consequence, a certain theoretical significance level on the vertical axis can be related to a particular empirical type I error rate on the horizontal axis. For FAM-MDR (panel A) the curve lies slightly above the diagonal, indicating a rather conservative approach. For FAM-MDR* (panel B) and PGMDR (panel C) the curve lies far below the diagonal, pointing to an extremely liberal approach. Only when performing the PGMDR on simulated data with two functional (and no non-functional!) SNPs (panel D), the PP-plot looks acceptable, although rather conservative as well.

**Table 2:** Type I error rates under the general null hypothesis of no association.

		Corr.			No C.		
*p*	*h^2^*	F	F*	PG	F	F*	PG
0.1	0.3	0.0200	**0.5700**	**0.4450**	0.0250	**0.6725**	**0.4700**
	0.5	0.0275	**0.4550**	**0.5300**	0.0300	**0.5650**	**0.5300**
	0.8	0.0100	**0.4825**	**0.5950**	0.0175	**0.5450**	**0.4900**
0.25	0.3	0.0275	**0.5650**	**0.4750**	0.0250	**0.6650**	**0.5250**
	0.5	0.0225	**0.5325**	**0.4750**	0.0200	**0.6025**	**0.5200**
	0.8	0.0175	**0.5125**	**0.4800**	0.0150	**0.6000**	**0.4800**
0.3	0.3	0.0275	**0.5525**	**0.5400**	0.0275	**0.6475**	**0.5600**
	0.5	0.0175	**0.5625**	**0.5350**	0.0325	**0.6300**	**0.5600**
	0.8	0.0100	**0.4900**	**0.4750**	0.0100	**0.5250**	**0.5100**

Results are based on 400 simulated datasets for each setting for FAM-MDR and FAM-MDR* and 200 datasets for PGMDR. As an aid to interpretation the type I error rates 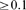 are indicated in bold. Abbreviations: Corr. = with main effects correction, No C. = without main effects correction, F = FAM-MDR, F* = FAM-MDR*, PG = PGMDR.

Second, we considered the simulated null data under the null hypothesis of no epistasis, in the presence of main effects. If under this null a two-locus model is identified (that is, a two-locus model is assessed significant), then a type I error has been made with regard to the null hypothesis of no interaction, and the significant result may be largely driven by main effects. Type I error rates for a variety of scenarios are given in Table 3. The analyses correcting for main effects have reasonable type I error rates, although also they tend to be rather conservative. This holds for both Models M27 and M170. The analyses not correcting for main effects have the tendency to give rise to an increased number of false positives. This increase is sometimes dramatic, going even up to 1. The estimated type I error rates point towards the importance of using analysis techniques that are able to appropriately account for important lower order effects. The observation that type I error rates increase with increasing values of 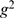, is to be expected. Indeed, when 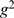 increases, also the main effects variance increases. Moreover, as the minor allele frequency 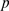 of the functional SNPs increases, type I error rates increase for Model M27 but decrease for Model M170. This can be explained by the relative importance of the main effects with varying 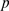 for the different models (Table 1). Note that for Model M170 and 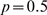 no results are stated because this situation corresponds to a pure epistasis scenario. Figure S1 (Supporting Information) gives a graphical illustration of the liberal type I error rates. Panel D shows results for the analysis not correcting for main effects on data generated under the null of no epistasis for model M27 and the extreme 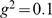. We observe that the probability-probability plot in this setting is perfectly horizontal at height 0. This extreme case corresponds to the fact that the type I error rate is 1 for this situation.

**Table 3:** Type I error rates of FAM-MDR under the null hypothesis of no epistasis.

		M27						M170			
		*p* = 0.1		*p* = 0.25		*p* = 0.5		*p* = 0.1		*p* = 0.25	
*g^2^*	*h^2^*	Corr.	No C.	Corr.	No C.	Corr.	No C.	Corr.	No C.	Corr.	No C.
0.01	0.3	0.05	0.09	0.02	0.07	0.01	**0.21**	0.03	**0.16**	0.01	0.01
	0.5	0.00	0.01	0.01	0.03	0.03	**0.16**	0.00	0.03	0.00	0.03
	0.8	0.03	0.04	0.01	0.05	0.02	0.06	0.01	0.02	0.00	0.01
0.02	0.3	0.01	**0.10**	0.03	**0.23**	0.03	**0.40**	0.03	**0.22**	0.07	0.07
	0.5	0.02	0.06	0.00	**0.17**	0.03	**0.35**	0.03	**0.19**	0.03	0.03
	0.8	0.00	0.03	0.01	**0.12**	0.03	**0.35**	0.01	**0.20**	0.01	0.00
0.03	0.3	0.03	**0.21**	0.01	**0.41**	0.01	**0.66**	0.01	**0.40**	0.05	0.08
	0.5	0.02	**0.16**	0.02	**0.38**	0.01	**0.68**	0.02	**0.33**	0.00	0.04
	0.8	0.01	0.09	0.01	**0.40**	0.02	**0.72**	0.01	**0.32**	0.02	0.03
0.05	0.3	0.03	**0.42**	0.02	**0.75**	0.02	**0.94**	0.01	**0.79**	0.06	**0.11**
	0.5	0.02	**0.26**	0.01	**0.82**	0.02	**0.96**	0.00	**0.82**	0.02	**0.13**
	0.8	0.01	**0.18**	0.02	**0.72**	0.01	**0.96**	0.01	**0.63**	0.01	0.09
0.1	0.3	0.03	**0.96**	0.02	**1.00**	0.02	**1.00**	0.00	**1.00**	0.01	**0.29**
	0.5	0.03	**0.84**	0.01	**0.99**	0.01	**1.00**	0.03	**0.99**	0.01	**0.34**
	0.8	0.00	**0.67**	0.02	**0.99**	0.00	**1.00**	0.03	**1.00**	0.01	**0.37**

Results are based on 100 simulated datasets for each setting. As an aid to interpretation the type I error rates 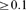 are indicated in bold. Abbreviations: Corr. = with main effects correction, No C. = without main effects correction.

**Figure 3:**
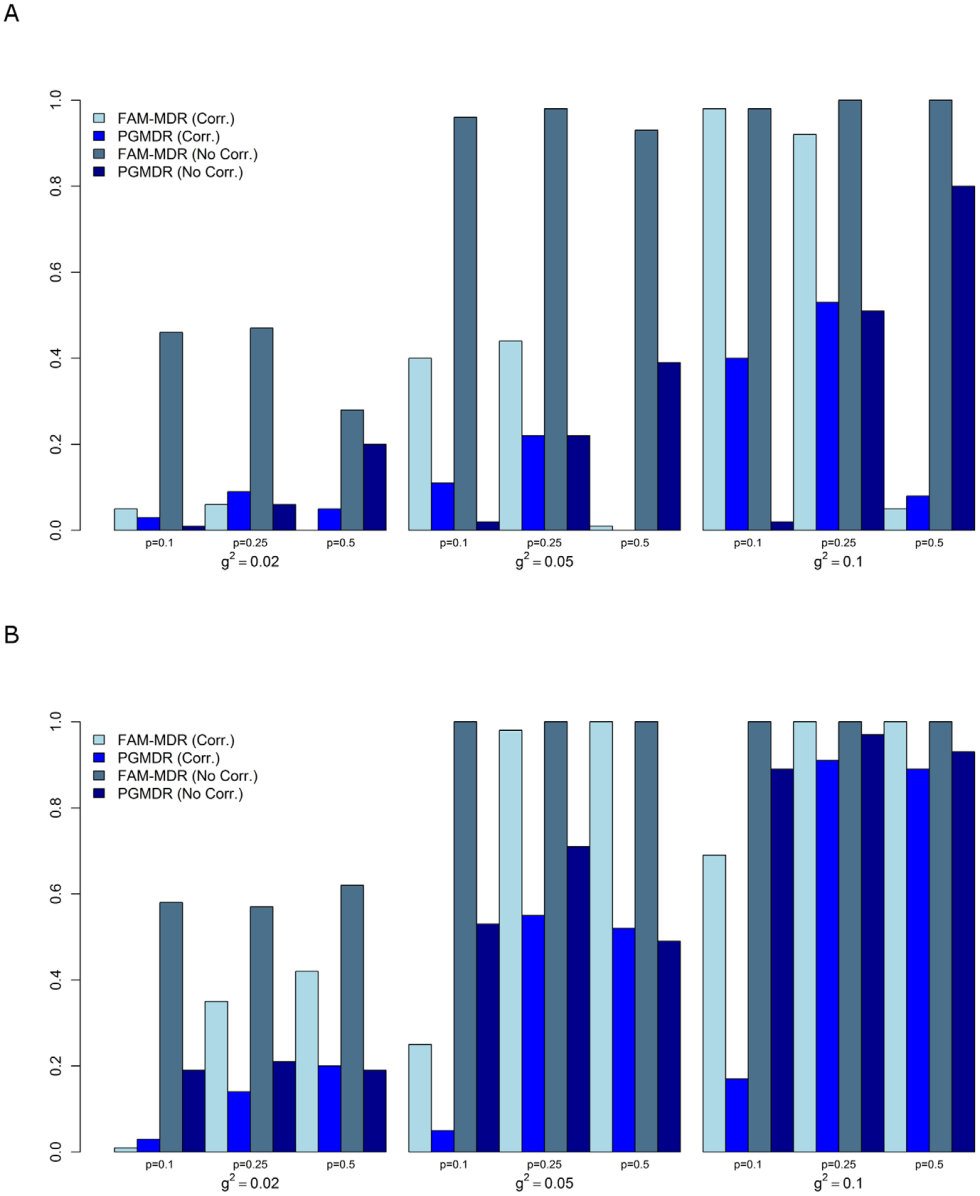
Power of FAM-MDR and PGMDR based on 100 replicates, with 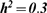. The different panels show results for M27 (A) and M170 (B). Abbreviations: Corr. = with main effects correction, No Corr. = without main effects correction.

Third, we considered simulated data under the alternative hypothesis of epistasis (possibly in the presence of main effects). For every simulation setting, empirical power was defined as the number of times the correct pair 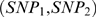 was selected and declared significant, divided by the number of simulated data sets. For PGMDR, it happened that two or even three models were reported by the software. In this event, the reported models had exactly the same cross-validation consistency, and we selected the model with the lowest permutation p-value. In the rare event that also the reported permutation p-values were tied, we made a random choice, except when the true functional model was among the reported results. In the latter case, we always selected the true functional model among the tied results. Table 4 gives an overview of the simulation results under a variety of alternative hypotheses. Figure 3 displays our findings graphically for 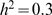. In the Supporting Information, we give additional power graphs for 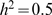 and 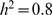, for Model M27 (Figure S2) and Model M170 (Figure S3), but these do not fundamentally differ from those for 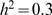. In general, FAM-MDR has systematically higher power than PGMDR, even with appropriate correction for multiple testing. Note that FAM-MDR* may well be the most powerful approach but, like PGMDR, it does not appropriately handle multiple testing problems. There are a few exceptions to these general observations. However, in these exceptional cases, the power gain of PGMDR is always very small, and the apparent better performance of PGMDR can just as well be due to sampling variability. Indeed, the standard error of a binomial proportion is about 0.05 for 100 simulated datasets. The results of Table 4 show that power estimates increase with increasing two-locus heritability 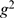.

**Table 4:** Power of FAM-MDR, FAM-MDR* and PGMDR.

			M27						M170					
			Corr.			No C.			Corr.			No C.		
*p*	*g^2^*	*h^2^*	F	F*	PG	F	F*	PG	F	F*	PG	F	F*	PG
0.1	0.01	0.3	0.03	0.17	0.03	0.19	0.40	0.01	0.01	0.10	0.01	0.16	0.36	0.06
		0.5	0.01	0.12	0.03	0.13	0.47	0.00	0.00	0.06	0.00	0.13	0.41	0.10
		0.8	0.02	0.19	0.00	0.09	0.42	0.01	0.01	0.14	0.00	0.08	0.35	0.04
	0.02	0.3	0.05	0.30	0.03	0.46	0.67	0.01	0.01	0.20	0.03	0.58	**0.79**	0.19
		0.5	0.05	0.38	0.02	0.43	0.68	0.01	0.03	0.20	0.01	0.49	**0.71**	0.01
		0.8	0.06	0.49	0.01	0.53	**0.82**	0.01	0.05	0.26	0.02	0.42	**0.73**	0.23
	0.03	0.3	0.12	0.67	0.05	**0.83**	**0.91**	0.01	0.05	0.30	0.04	**0.78**	**0.91**	0.33
		0.5	0.24	**0.72**	0.04	**0.87**	**0.97**	0.00	0.11	0.43	0.00	**0.90**	**0.98**	0.31
		0.8	0.32	**0.73**	0.02	**0.73**	**0.94**	0.01	0.15	0.48	0.01	**0.93**	**0.99**	0.32
	0.05	0.3	0.40	**0.90**	0.11	**0.96**	**0.99**	0.02	0.25	**0.73**	0.05	**1.00**	**1.00**	0.53
		0.5	0.48	**0.93**	0.10	**0.98**	**0.99**	0.02	0.30	**0.75**	0.03	**1.00**	**1.00**	0.55
		0.8	0.67	**0.95**	0.09	**0.98**	**1.00**	0.01	0.53	**0.90**	0.06	**1.00**	**1.00**	0.61
	0.1	0.3	**0.98**	**1.00**	0.40	**0.98**	**0.98**	0.02	0.69	**0.94**	0.17	**1.00**	**1.00**	**0.89**
		0.5	**0.96**	**1.00**	0.32	**1.00**	**1.00**	0.03	**0.80**	**0.97**	0.21	**1.00**	**1.00**	**0.95**
		0.8	**0.95**	**1.00**	0.28	**1.00**	**1.00**	0.00	**0.81**	**0.97**	0.13	**1.00**	**1.00**	**0.94**
0.25	0.01	0.3	0.01	0.06	0.02	0.14	0.29	0.04	0.07	0.29	0.11	0.21	0.49	0.13
		0.5	0.03	0.10	0.05	0.13	0.32	0.04	0.09	0.25	0.07	0.20	0.46	0.10
		0.8	0.00	0.13	0.05	0.08	0.31	0.02	0.15	0.51	0.06	0.23	0.58	0.07
	0.02	0.3	0.06	0.22	0.09	0.47	**0.70**	0.06	0.35	**0.74**	0.14	0.57	**0.82**	0.21
		0.5	0.04	0.13	0.06	0.32	0.61	0.06	0.40	**0.72**	0.21	0.60	**0.87**	0.29
		0.8	0.11	0.28	0.08	0.47	**0.72**	0.07	0.64	**0.95**	0.16	**0.88**	**0.97**	0.21
	0.03	0.3	0.14	0.38	0.12	**0.80**	**0.91**	0.08	0.65	**0.90**	0.20	**0.86**	**0.98**	0.27
		0.5	0.19	0.52	0.12	**0.78**	**0.88**	0.10	**0.77**	**0.95**	0.27	**0.92**	**0.94**	0.35
		0.8	0.16	0.45	0.05	**0.80**	**0.91**	0.05	**0.90**	**0.97**	0.24	**0.95**	**0.99**	0.32
	0.05	0.3	0.44	**0.80**	0.22	**0.98**	**0.98**	0.22	**0.98**	**1.00**	0.55	**1.00**	**1.00**	**0.71**
		0.5	0.53	**0.77**	0.19	**0.99**	**1.00**	0.20	**1.00**	**1.00**	0.48	**1.00**	**1.00**	0.58
		0.8	0.55	**0.80**	0.19	**1.00**	**1.00**	0.15	**0.98**	**1.00**	0.48	**1.00**	**1.00**	0.61
	0.1	0.3	**0.92**	**0.97**	0.53	**1.00**	**1.00**	0.51	**1.00**	**1.00**	0.91	**1.00**	**1.00**	**0.97**
		0.5	**0.97**	**1.00**	0.54	**1.00**	**1.00**	0.56	**1.00**	**1.00**	0.91	**1.00**	**1.00**	**0.95**
		0.8	**1.00**	**1.00**	0.47	**1.00**	**1.00**	0.51	**1.00**	**1.00**	0.89	**1.00**	**1.00**	**0.97**
0.5	0.01	0.3	0.00	0.02	0.00	0.08	0.21	0.07	0.02	0.27	0.04	0.17	0.41	0.06
		0.5	0.00	0.00	0.01	0.10	0.22	0.07	0.05	0.27	0.08	0.12	0.47	0.13
		0.8	0.00	0.01	0.02	0.05	0.18	0.07	0.27	0.57	0.07	0.36	0.61	0.10
	0.02	0.3	0.00	0.02	0.05	0.28	0.44	0.20	0.42	0.69	0.20	0.62	**0.83**	0.19
		0.5	0.00	0.05	0.02	0.29	0.42	0.16	0.44	0.69	0.18	0.60	**0.84**	0.20
		0.8	0.00	0.03	0.01	0.41	0.57	0.14	0.69	**0.90**	0.18	**0.77**	**0.91**	0.14
	0.03	0.3	0.02	0.08	0.03	0.57	0.65	0.22	**0.78**	**0.91**	0.31	**0.84**	**0.95**	0.33
		0.5	0.00	0.06	0.02	0.62	0.69	0.25	**0.93**	**0.96**	0.35	**0.93**	**0.99**	0.32
		0.8	0.02	0.10	0.02	0.69	**0.75**	0.24	**0.97**	**1.00**	0.27	**0.97**	**0.98**	0.28
	0.05	0.3	0.01	0.09	0.00	**0.93**	**0.93**	0.39	**1.00**	**1.00**	0.52	**1.00**	**1.00**	0.49
		0.5	0.02	0.11	0.02	**0.92**	**0.92**	0.45	**0.99**	**1.00**	0.48	**1.00**	**1.00**	0.46
		0.8	0.02	0.27	0.05	**0.97**	**0.97**	0.44	**1.00**	**1.00**	0.60	**1.00**	**1.00**	0.57
	0.1	0.3	0.05	0.46	0.08	**1.00**	**1.00**	**0.80**	**1.00**	**1.00**	**0.89**	**1.00**	**1.00**	**0.93**
		0.5	0.10	0.46	0.07	**1.00**	**1.00**	**0.79**	**1.00**	**1.00**	**0.95**	**1.00**	**1.00**	**0.95**
		0.8	0.26	0.68	0.05	**1.00**	**1.00**	**0.85**	**1.00**	**1.00**	**0.92**	**1.00**	**1.00**	**0.92**

Results are based on 100 simulated datasets for each setting. As an aid to interpretation the powers 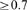 are indicated in bold. Abbreviations: Corr. = with main effects correction, No C. = without main effects correction , F = FAM-MDR, F* = FAM-MDR*, PG = PGMDR.

Our simulation data does not show a systematic trend with increasing total heritability 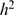. When correcting for main effects, the power decreases with minor allele frequency 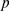 for model M27. The reverse is observed for Model M170. This can be explained by the proportion 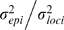 of the epistatic variance to the total two-locus variance, varying with 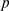 for the two models in a different way, as given in Table 1. For example, for Model M27 and 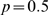, in which case the ratio 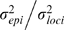 is only 0.14, the power is very low. For the analysis without correction for main effects, power is less dependent on 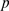 and high power levels are probably indicative for excessive type I error rates due to lower order effects signaling through to the higher order models. Correcting for main effects generally gives rise to lower power estimates than not correcting for main effects (Table 4 and Figure 3). There are two reasons for this. First, the epistatic variance 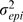 (targeted by an analysis with correction for main effects) is generally smaller than the total two-locus variance 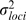 (which is the focus of an analysis without correction for main effects). Consequently, larger sample sizes are needed to enable epistasis detection. Second, there is some efficiency loss because every main effect corrected for contributes two degrees of freedom (as main effects were coded as co-dominant effects). That there is a price to pay for estimating additional parameters can most clearly be deduced from the results for Model M170 and 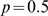 (pure epistasis), for which we still observe the reduction in power for the analysis with main effects corrections over an uncorrected analysis. Another example is that for Model M27 with 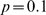 the analyses without main effects corrections 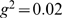 have higher power than the corrected analyses for 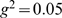, although in the first case 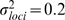 and in the second case 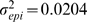.

### Amish Family Diabetes Study

Table 5 shows the results of epistasis screening with FAM-MDR and PGMDR on the split pedigree data, and of FAM-MDR on the multi-generational pedigree data, both with and without correction for co-dominant main effects of *rs1884614* in *HNF4A*, *rs2275703* and *rs617698* in *CASQ1* and *rs1029629* in *ADIPOR2*. Without correction for main effects, FAM-MDR on the cut-pedigree data finds a significant two-locus model involving *rs2275703* in *CASQ1* and *rs1501299* in *APM1*. When performing a main effects correction, the same best interaction model is identified. However, the interaction is significant only at the 10% significance level, suggesting that the two-locus model is to some extent driven by the main effect of *rs2275703*. PGMDR without main effects correction does not lead to a significant interaction. With correction for main effects, PGMDR reports a different near-significant two-locus model involving *rs2275703* in *CASQ1* and *rs1028583* in *HNF4A*. FAM-MDR on the original data, with or without correction for main effects, does not yield any significant two-locus models. Possible explanations for these contradicting results are reviewed in the Discussion section.

**Table 5:** Epistasis analyses of Amish Family Diabetes Study data.

		Corr.		No C.	
		Model	P-value	Model	P-value
Orig.	F (F*)	*rs2275703* in *CASQ1*; *rs1501299* in *APM1*	0.619 (0.008)	*rs2275703* in *CASQ1*; *rs1501299* in *APM1*	0.280 (0.005)
Split	F (F*)	*rs2275703* in *CASQ1*; *rs1501299* in *APM1*	0.070 (0.001)	*rs2275703* in *CASQ1*; *rs1501299* in *APM1*	0.014 (<0.001)
Split	PG	*rs2275703* in *CASQ1*; *rs1028583* in *HNF4A*	0.059	rs1029629 in *ADIPOR2*; rs2425637 in *HNF4A*	0.303

Main effects corrections adjust the analyses for *rs1884614* in *HNF4A*, *rs2275703* and *rs617698* in *CASQ1*, and *rs1029629* in *ADIPOR2*. Abbreviations: Corr. = with main effects correction, No C. = without main effects correction, Orig. = original data, Split = split pedigree, F = FAM-MDR, F* = FAM-MDR*, PG = PGMDR.

## Discussion

The objectives of this paper were to introduce a new family-based and flexible epistasis detection analysis method, FAM-MDR, which is based on multifactor dimensionality reduction of multi-locus genotypes, and to compare it to the current state-of-the art MDR methodology for families, PGMDR. Although principles of the initial MDR approach are adopted in FAM-MDR, there are some clear differences. These include an alternative way to identify risk categories associated with multi-locus genotypes, the flexibility to use any outcome type, the possibility to correct for lower order effects, covariates or confounding factors, the possibility to assess significance of multiple higher-order interaction models. Since model selection is not based on evaluating prediction accuracy but on testing associations, FAM-MDR does not involve computationally intensive cross-validation steps.

FAM-MDR consists of two parts. In part I, residuals are derived from a polygenic model, removing additive polygenic effects and possibly important lower-order effects or confounding factors. These residuals are subsequently considered as new traits for the second part of FAM-MDR. In Part II, the familial correlation-free residuals are submitted to the MB-MDR algorithm and either the best model (considered in this manuscript), or multiple epistasis models are checked for their significance. In contrary to first implementations of the MB-MDR algorithm, no simulation-based null distributions are derived to assess significance, but a permutation-based strategy is adopted. Under the assumption of familial correlation-free traits in FAM-MDR Part II, permutation-based p-values for the best model can easily be derived by randomly permuting the traits. This is in contrast to PGMDR's implementation of a permutation strategy in that for PGDMR families are considered as the permutation units.

The method of removing familial-correlation structure is not new. The GRAMMAR approach of Aulchenko et al. [39] and Amin et al. [42] also used this idea in the context of rapid genomewide main effects analysis. For multivariate traits and unrelated individuals, similar principles of first removing trait correlations and then submitting derived residuals to MB-MDR can be adopted. This is work in progress and is particularly useful when measurements over time are available. Exploiting the time-relatedness of phenotypic measurements may in part compensate for the large sample sizes needed to detect epistasis in genomic studies.

The Current implementation of FAM-MDR is not scalable to GWAS. An efficient C++ implementation and a code version for parallel analyses are on their way. First simulations indicate that these enhancements will make GWAS feasible. For now, when large-scale genomic screenings are performed with thousands of markers, since FAM-MDR Part I is preparatory for subsequent association analysis, FAM-MDR can include a pre-selection step of good candidates of markers for epistasis analysis. These candidates may be selected on the basis of information theoretic measures [57], [58] using information about the trait under investigation; or on the basis of evidence from other data, i.e. using external and independent information [59], e.g. omics analyses. Depending on the strategy, an additional correction for data snooping in the pre-screening step may be required to control type I error rates.

Also, a Part III can be added to a FAM-MDR analysis to interpret the identified epistasis models. This is an important step of the analysis and may or may not involve deriving good estimates of the significant effects. Special care needs to be taken when carrying out this step, in order not to be the victim of the so-called “winner's curse” [60], [61].

Currently, MB-MDR and FAM-MDR are based on Wald statistics, whereas GMDR and PGMDR make use of score statistics. First, the score test is computationally more advantageous because it only needs parameter estimates under the null whereas the Wald test needs parameter estimates under the alternative and the likelihood ratio test needs both. Second, even though the three tests are asymptotically equivalent, the score test is the most powerful of the three when the true parameter is close to the null value. In line with the first release of an MB-MDR R package for unrelated individuals, Wald statistics were implemented in the first version of the FAM-MDR software. In the future, score statistics as well as robust non-parametric statistics will be offered as additional options in FAM-MDR. One of the major results that our simulation study highlights is that PGMDR is too liberal in identifying epistasis models. This is due to the inadequate correction for multiple testing, implemented in the PGDMR software to date (Figure 2; Panel C). In contrast, FAM-MDR correctly deals with multiple testing and consequently leads to appropriate type I error rates (Figure 2; Panel A). In effect, FAM-MDR is rather conservative, which is a property inherited from the GRAMMAR approach it is built on. Indeed, while first removing polygenic effects (FAM-MDR Part I), an over-correction may take place, resulting in power loss and conservatism to identify remaining genetic association signals. Improvements to FAM-MDR that can remove this artifact are on the way. A second result is that, generally speaking, FAM-MDR has optimal power over PGMDR in virtual all considered simulation scenarios. In addition, we have indicated that occasional better achieved performance of PGMDR in terms of power is probably attributable to sampling variability. Also note that when computing the PGMDR power estimates in our simulation study, in case of a tie we gave advantage to the model with the functional SNP pair.

A third important result is the influence of correcting for lower-order effects when searching for significant epistatic interactions. As was also pointed out by Calle et al. [40], MDR-like analysis that does not account for important marginal effects is prone to report false higher-order interactions, containing the significant lower-order effects not accounted for. Although PGMDR accommodates covariate adjustment, more work is needed to enhance flexible implementation. FAM-MDR code is currently available as an R-script, in which covariate adjustments are easily incorporated in the model statement of the polygenic function. More work is needed though to develop a genuine screening strategy to search for optimal models, starting from important main effects and ending with higher-order interaction models beyond the two-way interactions considered in this work, with the maximum order pre-defined by the user. This is future research, since our simulations have shown that it is of utmost importance to adjust for previous significant findings when moving to the search space of interactions of the next order. Since the way lower-order interactions are accounted for is part of a parametric paradigm, the coding of these effects needs careful reflection. When lower-order effects are important, a correction is warranted. When lower-order effects are not important or not adequately coded, over-adjusting for lower-order effects may lead to there being virtually no variation left with which to identify higher-order interactions. Hence, in reality the balance between necessary corrections for important main effects and avoiding over-correction needs to be considered to optimize the performance of any epistasis detection method.

The current FAM-MDR implementation is only valid under the assumption of no population structure, and our simulations assume a homogeneous population. By using both between- and within-family association – in contrast to PGMDR that uses only within-family association – FAM-MDR gains power but this comes at the price of sacrificing the built-in protection against spurious results if population structure is present. A possible solution to this problem lies in the use of Genomic Control [62]. Although PGMDR is a flexible tool to handle binary or continuous outcome types, and accommodates covariate adjustment, our application to real-life data has revealed some important shortcomings that impact the power of a study. These include a rather inefficient use of available information and the inability to analyze complex and extended pedigrees with the present PGMDR implementation.

Regarding missing genotypes, PGMDR discards families entirely when data on one SNP are missing. Without minimizing the need to also improve FAM-MDR's handling of missing data (whether at the genotype or phenotype level), not being able to account for the full complexity of a pedigree is certainly a drawback of PGMDR. We believe that methods that can accommodate mixed study designs will become more and more important due to the increasing practice of combining data from different groups in consortia collaborations. FAM-MDR flexibly deals with both unrelated and related individuals in the same analysis whereas PGMDR excludes unrelated individuals, hereby reducing the power of the association analysis.

Multi-generational pedigrees may provide more information on inheritance patterns observed on genotype data, which improves the quality of family-based tests of association. Due to limitations of the PGMDR software, only FAM-MDR analysis was applied to the extended pedigree data as such. With (or without) FAM-MDR correction for main effects of *rs1884614* in *HNF4A*, *rs2275703* and *rs617698* in *CASQ1*, and *rs1029629* in *ADIPOR2*, no evidence for a significant interaction (or two-locus model) was found (Table 5). Weak interactions between variations at these loci in contributing to the 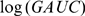 phenotype may still exist, but if so, FAM-MDR was not able to identify them. In this context it is important to note that FAM-MDR – like GRAMMAR – could be rather conservative, especially for larger extended pedigrees [42]. On the other hand, the (nearly) significant findings for the simplified pedigree (Table 5) may potentially be false positive results driven by both the artificial increase in sample size and by not appropriately accounting for family structure. Indeed, McArdle et al. [63] showed that the type I error of detecting SNP main effects is elevated when family structure is ignored, and more dramatically with increasing trait heritability, which may naturally extend to (interactive) two-locus models as well. Lack of appropriate accounting for multiple testing (PGMDR and FAM-MDR*) may also increase the likelihood of observing spurious associations. This is clearly seen for FAM-MDR* and to some extent also for PGMDR (Table 5).

Improving the ability to detect gene-gene interactions in family studies of complex disease may prove critical in identifying the underlying sources of observed heritability. While it is apparent that type 2 diabetes mellitus (T2DM) is polygenic, the genetic sources of the observed heritability have yet to be completely identified [64], and may include still unobserved gene-gene interactions. Detecting interactions between genes associated with T2DM has thus far been very challenging, due to a paucity of powerful statistical methods and study datasets with adequate sample sizes [65]. Several studies that have examined interactions between single variants in different genes have shown mixed evidence of two-way interactions in T2DM and T2DM-related traits like obesity and insulin resistance. Using an approach which conditioned on linkage in one region to identify evidence of linkage elsewhere, a linkage study of T2DM in Mexican Americans from Starr County, Texas, identified the interaction of genes on chromosomes 2 (*CAPN10* (Calpain 10), then *NIDDM1*) and 15 (near *CYP19* (Cytochrome P450, family 19, or Aromatase)) in contributiing to T2DM susceptibility [66]. Association studies investigating interactions between variants of the Beta-3 adrenergic receptor (*ADRB3*) and Uncoupling protein 1 (*UCP1*) genes observed in weak [67] to no [68] effects on weight gain and insulin resistance in Finnish and Danish populations, respectively. A study of Type II iodothyronine deiodinase (*DIO2*) and *ADRB3* polymorphisms showed a synergistic effect on an increased BMI, suggesting an interaction between these two common gene variants [69], while a study of intestinal fatty acid binding protein 2 (FABP2) and ADRB3 showed no interaction on levels of fasting plasma glucose or measures of insulin resistance [70]. In a study of Mexican-American families participating in the population-based San Antonio Family Heart Study [71], the combined presence of common variants of Peroxisome proliferator-activated receptor gamma (PPARγ) and ADRB3 are correlated with significantly higher BMI, insulin, and leptin levels than the presence of the PPARγ variants alone. Yet another study [72] examined two-locus interactions among 23 loci in T2DM candidate genes in the risk of T2DM, and found a significant interaction between variants in the Uncoupling protein 1 (UCP2) and PPARγ genes. Identification of novel interactions and further confirmation of observed interactions may be critical in characterizing the genetic risk factors for T2DM and many other complex disease that remain among the unidentified components of the heritability of these diseases, and may have practical application in the identification of individuals who may belong to groups at high risk of disease who can benefit from preventive care.

In conclusion, FAM-MDR – unlike PGMDR – is able to handle complex and large pedigrees with additional unrelated individuals. In fact, FAM-MDR analysis on split pedigree data should not be trusted because it might lead to overly optimistic results. On the other hand, as pedigree size increases, the inherent conservative nature of FAM-MDR could become more pronounced. Finally, PGMDR results – and in fact also GMDR results – are too liberal as no correction for multiple testing is carried out.

## Supporting Information

Figure S1 *Probability-probability plots for FAM-MDR analyses under the null hypotheses of no association and no epistasis*. The situation considered is p = 0.5 and h^2^ = 0.3. Analyses are performed both with and without correction for main effects. Results are based on 100 replicates. Panels A and B show results for data generated under the null of no association, whereas panels C and D consider data generated under the null hypothesis of no epistasis, for model M27 and with g^2^ = 0.1. Panels A and C show results for analysis with correction for main effects, panels B and D without.
(0.50 MB TIF)

Figure S2 *Additional power results for model M27, based on 100 replicates*. Panels A and B show results for h^2^ = 0.5 and h^2^ = 0.8 respectively. Abbreviations: Corr. = with main effects correction, No Corr. = without main effects correction.
(1.54 MB TIF)

Figure S3 *Additional power results for model M170, based on 100 replicates*. Panels A and B show results for h^2^ = 0.5 and h^2^ = 0.8 respectively. Abbreviations: Corr. = with main effects correction, No Corr. = without main effects correction.
(1.53 MB TIF)
