# Supplementary figures and images for: FAM-MDR: A Flexible Family-Based Multifactor Dimensionality Reduction Technique to Detect Epistasis Using Related Individuals
Source: PLoS One. 2010 Apr 22;5(4):e10304. doi: 10.1371/journal.pone.0010304 (PMC2858665; doi:10.1371/journal.pone.0010304)

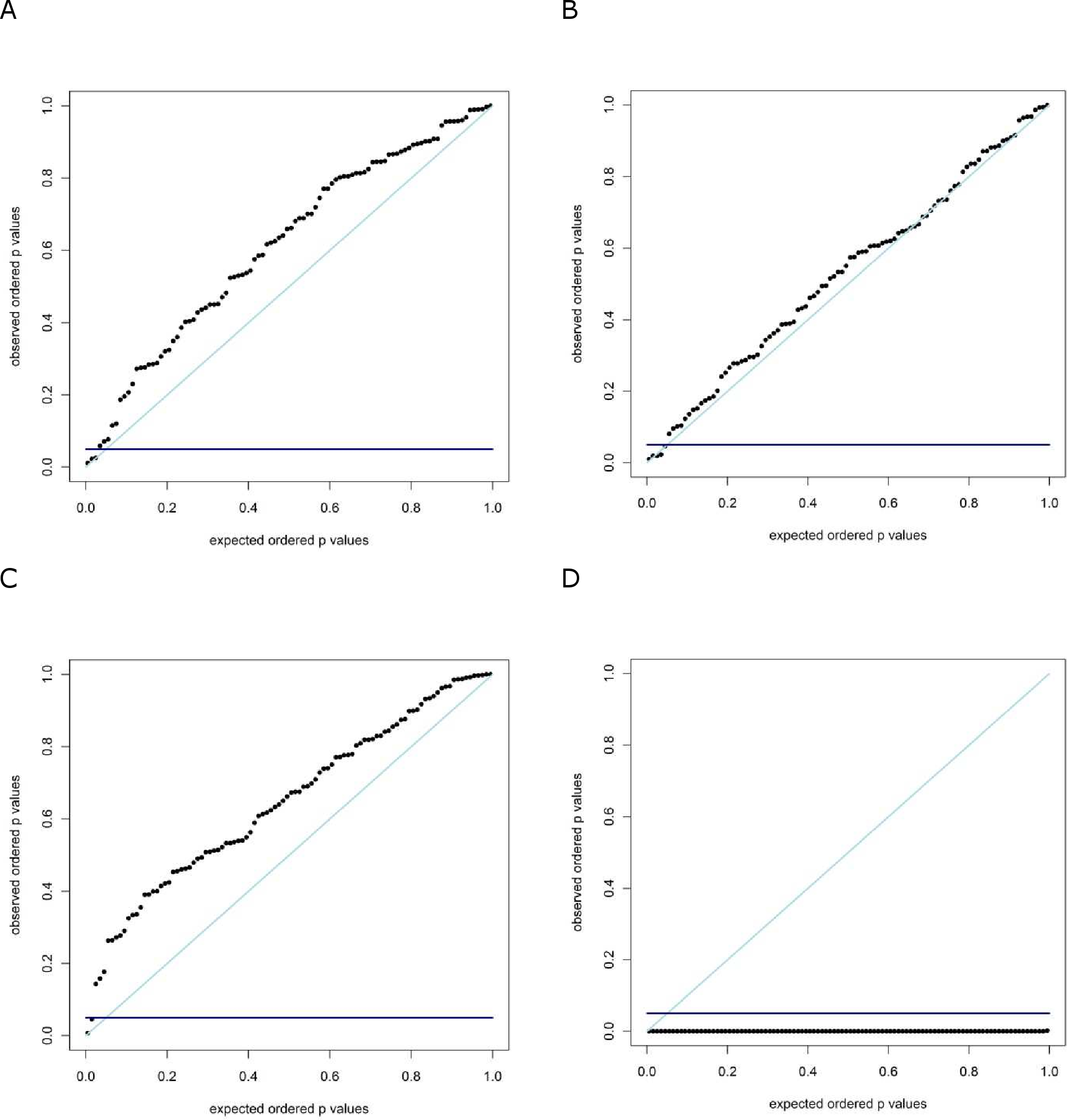

Supplement: Figure S1 — Probability-probability plots for FAM-MDR analyses under the null hypotheses of no association and no epistasis. The situation considered is p = 0.5 and h2 = 0.3. Analyses are performed both with and without correction for main effects. Results are based on 100 replicates. Panels A and B show results for data generated under the null of no association, whereas panels C and D consider data generated under the null hypothesis of no epistasis, for model M27 and with g2 = 0.1. Panels A and C show results for analysis with correction for main effects, panels B and D without. (0.50 MB TIF) [file pone.0010304.s001.tif]

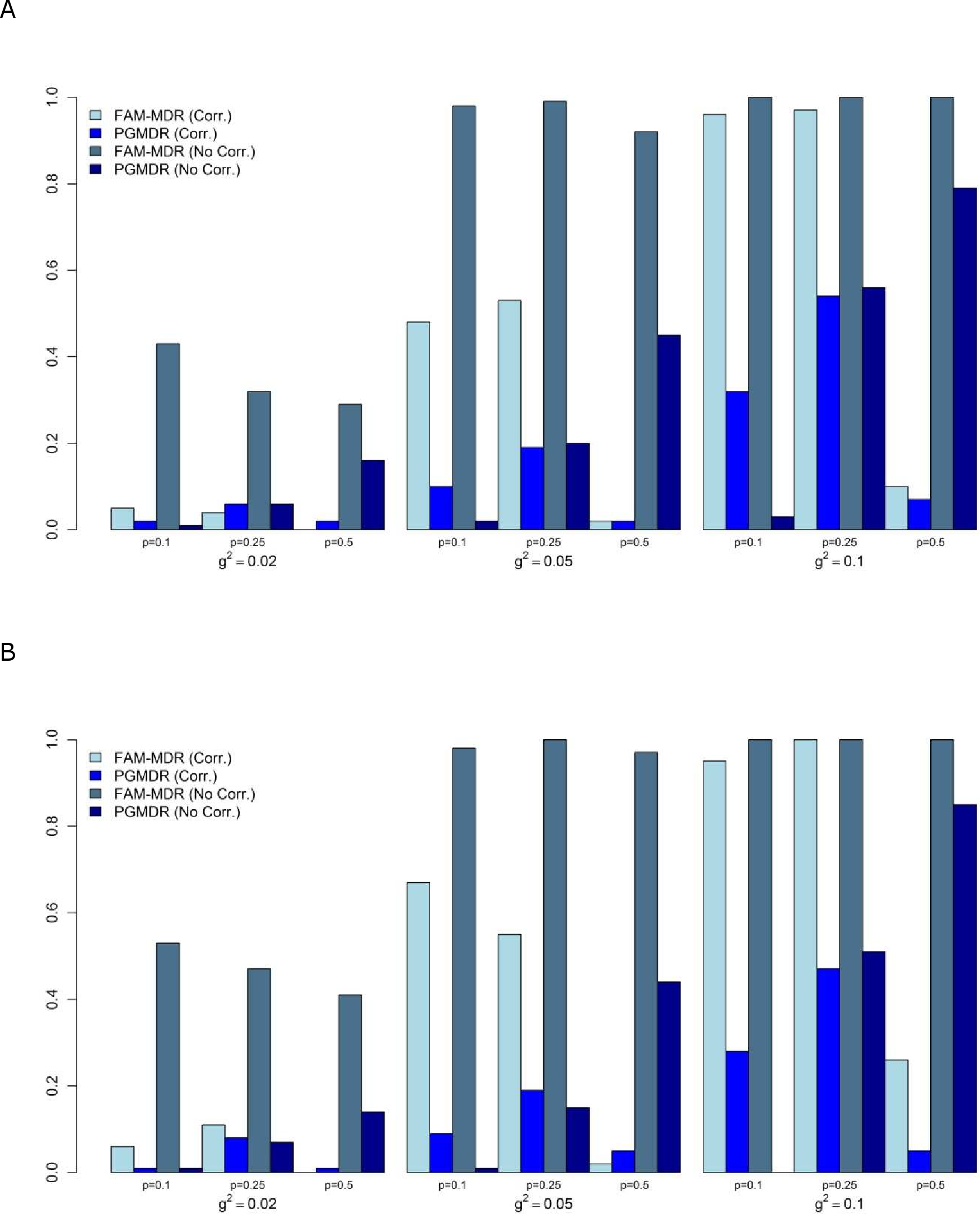

Supplement: Figure S2 — Additional power results for model M27, based on 100 replicates. Panels A and B show results for h2 = 0.5 and h2 = 0.8 respectively. Abbreviations: Corr. = with main effects correction, No Corr. = without main effects correction. (1.54 MB TIF) [file pone.0010304.s002.tif]

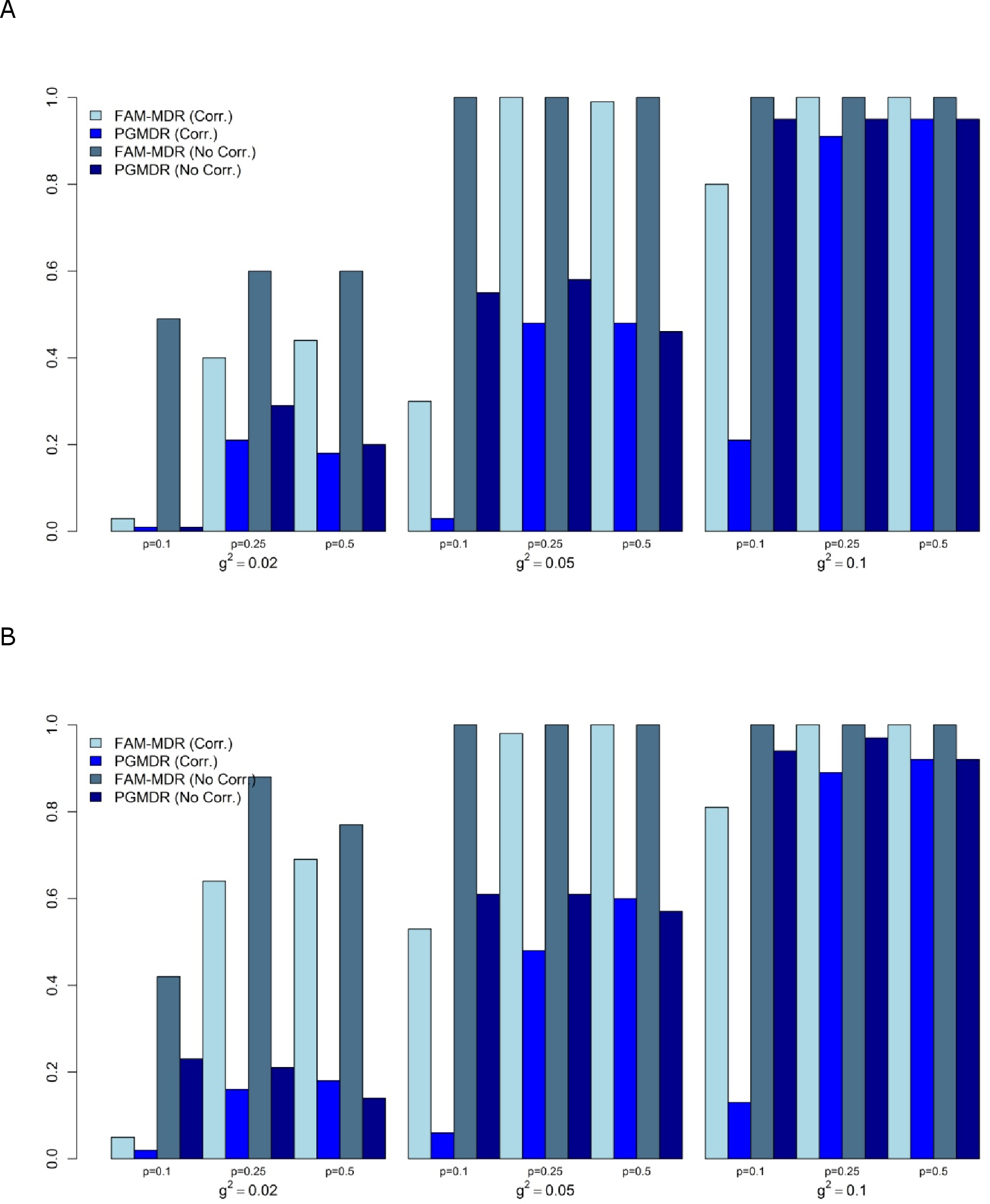

Supplement: Figure S3 — Additional power results for model M170, based on 100 replicates. Panels A and B show results for h2 = 0.5 and h2 = 0.8 respectively. Abbreviations: Corr. = with main effects correction, No Corr. = without main effects correction. (1.53 MB TIF) [file pone.0010304.s003.tif]
